# Genomic and transcriptomic profiles associated with response to eribulin and nivolumab combination in HER-2-negative metastatic breast cancer

**DOI:** 10.1007/s00262-024-03782-7

**Published:** 2024-08-06

**Authors:** Changhee Park, Koung Jin Suh, Se Hyun Kim, Kyung-Hun Lee, Seock-Ah Im, Min Hwan Kim, Joohyuk Sohn, Jae Ho Jeong, Kyung Hae Jung, Kyoung Eun Lee, Yeon Hee Park, Hee-Jun Kim, Eun Kyung Cho, In Sil Choi, Seung-Jae Noh, Inkyung Shin, Dae-Yeon Cho, Jee Hyun Kim

**Affiliations:** 1 Division of Hematology-Oncology, Department of Internal Medicine, Seoul National University Bundang Hospital, 82, Gumi-Ro, Bundang-Gu, Seongnam, 13620 Republic of Korea; 2 Department of Internal Medicine, Seoul National University Hospital, Cancer Research Institute, Seoul National University, College of Medicine, Seoul, Republic of Korea; 3 Division of Medical Oncology, Department of Internal Medicine, Yonsei Cancer Center, Yonsei University College of Medicine, Seoul, Korea; 4 Department of Oncology, Asan Medical Center, University of Ulsan College of Medicine, Seoul, Korea; 5 Department of Hematology and Oncology, Ewha Womans University Hospital, Seoul, Korea; 6 Hematology-Oncology, Samsung Medical Center Sungkyunkwan University School of Medicine, Seoul, Korea; 7 Department of Internal Medicine, Chung-Ang University College of Medicine, Seoul, Korea; 8 Division of Oncology, Department of Internal Medicine, Gil Medical Center, Gachon University College of Medicine, Incheon, Korea; 9 Department of Internal Medicine, Seoul Metropolitan Government Seoul National University Boramae Medical Center, Seoul, Korea; 10 PentaMedix Co., Ltd, Seongnam, Korea

**Keywords:** Immunotherapy, Eribulin, HER-2-negative metastatic breast cancer, Genomics, Transcriptomics, Biomarker

## Abstract

**Background:**

Biomarkers for predicting response to the immunotherapy and chemotherapy combination in breast cancer patients are not established. In this study, we report exploratory genomic and transcriptomic analyses of pretreatment tumor tissues from patients enrolled in phase II clinical trial of a combination of eribulin and nivolumab for HER-2-negative metastatic breast cancer (MBC) (KORNELIA trial, NCT04061863).

**Methods:**

We analyzed associations between tumor molecular profiles based on genomic (*n* = 76) and transcriptomic data (*n* = 58) and therapeutic efficacy. Patients who achieved progression-free survival (PFS) ≥ 6 months were defined as PFS6-responders and PFS6-nonresponders otherwise.

**Findings:**

Analyses on tumor mutation burden (TMB) showed a tendency toward a favorable effect on efficacy, while several analyses related to homologous recombination deficiency (HRD) indicated a potentially negative impact on efficacy. Patients harboring *TP53* mutations showed significantly poor PFS6 rate and PFS, which correlated with the enrichment of cell cycle-related signatures in PFS6-nonresponders. High antigen presentation gene set enrichment scores (≥ median) were significantly associated with longer PFS. Naïve B-cell and plasma cell proportions were considerably higher in long responders (≥ 18 months).

**Interpretation:**

Genomic features including TMB, HRD, and *TP53* mutations and transcriptomic features related to immune cell profiles and cell cycle may distinguish responders. Our findings provide insights for further exploring the combination regimen and its biomarkers in these tumors.

**Supplementary Information:**

The online version contains supplementary material available at 10.1007/s00262-024-03782-7.

## Introduction

Although breast cancers are thought to be less immune-sensitive, clinical trials of chemotherapy combined with anti-programmed cell death 1 or anti-programmed cell death ligand-1 (PD-L1) immune checkpoint inhibitors atezolizumab or pembrolizumab in triple-negative breast cancer (TNBC) have shown favorable efficacy, making these combinations the standard of care for TNBC [1, 2]. These pioneering trials were followed by comprehensive investigations on immunotherapy combinations for breast cancer.

The combination of eribulin plus immune checkpoint inhibitors has received research attention. In preclinical studies, eribulin promoted antitumor-immune tumor microenvironments by remodeling the tumor vascular bed, decreasing tumor hypoxia, and inhibiting immune evasion [3]. As eribulin has been approved for use in metastatic breast cancer (MBC) [4], it may be a good partner for immune checkpoint inhibitor combinations for breast cancers. The first clinical trial of eribulin plus pembrolizumab, reported by Tolaney et al. [5], demonstrated potential antitumor effects in patients with TNBC. Subsequent studies reported the results of a randomized clinical trial of eribulin plus pembrolizumab or placebo for hormone-positive breast cancer (HRpos), showing no outcome improvement in the pembrolizumab combination group [6]. We also performed the combination of eribulin plus nivolumab for HRpos and TNBC [7]. Notably, subset of TNBC and HRpos patients showed prolonged response to eribulin plus immune checkpoint inhibitors in these clinical trials.

To determine the subset of patients who would show prolonged response to the treatment is pivotal. However, previous clinical trials have shown that PD-L1 immunohistochemistry expression was not significantly associated with the effectiveness of the eribulin plus immunotherapy combination regimen [5–7]. Therefore, further biomarker analyses are required to identify potential responders. The exploratory biomarker analysis of a randomized clinical trial by Tolaney et al. [8] showed that subgroups of patients with HRpos might benefit from pembrolizumab combination therapy. In this study, patients with high tumor mutation burden (TMB), low tumor purity, high immune infiltration, high antigen presentation (AP) expression signatures, and low estrogen response expression signatures showed favorable responses.

Here, we describe an exploratory biomarker analysis using next-generation sequencing (NGS) of pretreatment tumor tissues to evaluate the genomic and transcriptomic profiles associated with the response to the eribulin and nivolumab combination in patients with human epidermal growth factor receptor-2 (HER-2) negative breast cancer. We aim to explore such potential biomarker to determine the subgroup of patients who would respond to the treatment, to provide insights for further exploring the combination regimen and its biomarkers in these tumors.

## Materials and methods

### Study design

This study was an exploratory biomarker analysis using pretreatment formalin-fixed, paraffin-embedded (FFPE) tumor tissues from patients enrolled in a phase II multicenter clinical trial of eribulin plus nivolumab in patients with HER-2-negative MBC (KORNELIA trial, ClinicalTrials.gov Identifier: NCT04061863) [7]. The inclusion and exclusion criteria are described detailed in the previous literature. Briefly, we included HER-2-negative advanced breast cancer treated with anthracycline and/or taxane, which may have been delivered in either the neoadjuvant, adjuvant, or metastatic setting and experienced disease progression on or after taxane-based chemotherapy in the metastatic setting. The patient must have had less than three prior lines of cytotoxic chemotherapy for metastatic disease. Endocrine therapy was not counted as a prior line of treatment. Patients who previously received eribulin or any immune checkpoint inhibitor were excluded. The primary and secondary objectives were the 6-month progression-free survival (PFS6) rate and PFS, respectively.

### Clinical outcome parameters

For consistency with the previously published original trial, we focused on the primary objective of the trial: PFS6 rate, which is suggested as a surrogate endpoint in immune checkpoint inhibitor trials [9]. We divided patients according to the PFS6 criterion; patients who achieved PFS ≥ 6 months were grouped as PFS6-responders, otherwise as PFS6-nonresponders. For immune signature analysis, we also identified long responders as patients who achieved PFS ≥ 12 months.

### DNA and RNA extraction

Genomic DNAs and total RNAs were extracted from FFPE specimen using the Allprep DNA/RNA FFPE kit (Qiagen) following the manufacturer’s instructions. Genomic DNAs were extracted from peripheral blood using QIAamp DNA Blood Mini Kit (Qiagen). Both concentration and quality of all the isolated DNA and RNA samples were measured and checked with Qubit (Thermo Fisher Scientific) and Tapestation 4150 (Agilent Technologies).

### Whole-exome sequencing

Whole-exome libraries were built up using Twist Core Exome kit (Twist Bioscience). Briefly, FFPE-DNA was fragmented to 250–350 bp using a Covaris M220 ultrasonicator (Covaris). Hundred nanograms of fragmented DNA was end-repaired and dA-tailed using Twist Library Preparation kit following the manufacturer’s instructions. Libraries of gDNA extracted from blood were prepared in the same manner using 50 ng input.

Twist combinatorial dual (CD) Index adapters or Twist Universal adapters were ligated to repaired fragments, and the ligated fragments were amplified using PCR. The ligated fragments with CD index adapters were amplified for 10 PCR cycles. In the case of Universal adapter ligates, 7 PCR cycles were performed using Unique Dual Indexed (UDI) primer sets. Four to eight individual libraries were pooled to total mass of 1500 ng. The pool was hybridized at 60 °C for 4 h using Twist Human Core Exome panel. The exome library was amplified for 7 PCR cycles using KAPA HiFi HotStart ReadyMix (KAPA Biosystems).

The qualification and quantification estimations for each library were done after the last purification using Qubit assay (Thermo Fisher Scientific) and Tapestation system (Agilent Technologies). After normalization, the libraries were sequence on NextSeq 550Dx machines (Illumina) in paired-end 2 × 150 bp.

### RNA sequencing

RNA libraries were prepared using Illumina TruSeq Stranded Total RNA Library Prep Gold kit (Illumina) following manufacturer’s instructions starting with 100 ng to 1 ug total RNA. First, ribosomal RNAs were removed from total RNA before proceeding to the cDNA synthesis. rRNA-depleted RNAs were fragmented and converted cDNA with reverse transcriptase. The resulting cDNAs were converted to double stranded cDNAs and subjected to end-repair, A-tailing, and adapter ligation. The constructed libraries were amplified using 15 cycles of PCR. Libraries were quantified and qualified using Qubit (Thermo Fisher Scientific) and Tapestation 4150 (Agilent Technologies).

The libraries were sequenced by 150-bp paired-end reads on NovaSeq sequencer (Illumina).

### WES data processing

Whole-exome sequencing data were generated for 76 paired (tumor-normal) samples using the NextSeq550Dx machine in 2 × 150 bp mode (300 cycles).More than 5 gigabases (Gbs) of raw output were generated for peripheral blood normal samples (median 6.63 Gbs, ~ 180x) and 10Gbs for tumor FFPE samples (median 10.5 Gbs, ~ 300x). Raw sequencing data were preprocessed using Cutadapt v2.8 to remove adapter sequences and trim low-quality reads with the default option except –minimum-length 30. Trimmed reads were then aligned to the human reference genome hg19 using BWA-MEM (v0.7.17), and the aligned sequences were sorted using SAMtools (v1.10) and deduplicated to remove potential PCR artifacts using MarkDuplicates (GATK v4.1.4.0). In addition, WES reads were realigned and base quality scores were recalibrated according to GATK best practice to minimize erroneous variant calls.

### RNA data processing

Total RNA sequencing was also performed on 58 tumor FFPE samples with in 2 × 150 bp mode (300 cycles). More than 50 million reads were generated for each of 58 tumor FFPE samples (median 77.1 million reads, 11.64 Gbs). Raw reads were aligned to the hg19 genome using STAR aligner (v2.7.1) with default parameters after removing adapter sequences and trimming low-quality reads. Preprocessing and removal of duplicates were performed as previously described for whole-exome sequencing methods. Aligned reads were then counted using HTseq (v0.11.3), which analyses the expression levels of transcripts overlapping their exons for each gene. Using these read counts, we calculate FPKM (Fragments Per Kilobase of transcript per Million mapped reads) values for transcripts, which were log2 transformed and normalized for comparison between cohort expression profiles.

We also used TPM (Transcripts Per Million) values for the following analysis. For TPM normalization, we followed the method of Wagner et al. [10]. Briefly, we first calculated the RPK (Reads Per Kilobase) values by dividing the raw HTseq counts by the length of each gene in kilobases. We then added all of the RPK values for whole transcriptome in the sample and divided it by 1,000,000 to get the scaling factor per million. Finally, we obtained TPM values for each transcript by dividing each RPK value by the scaling factor.

### Variant calling

We utilized SomaticSeq software (v3.4.0), which improves the accuracy of somatic mutation calls by merging the results of multiple variant callers. In this study, we implemented 8 variant callers: Mutect2 (v4.0.5), VarScan2 (v2.3.7), VarDict (v1.7.0), LoFreq (v2.1.3.1), Strelka (v2.9.5), JointSNVMix2 (v0.7.5), SomaticSniper (v1.0.5), and MuSE (v1.0). Variants were annotated using Ensembl Variant Effect Predictor (VEP, v107) and the annotated information was parsed using an in-house Python script. Somatic variant calls satisfying filtering options described below were saved for further analysis; read depth from both normal and tumor is greater than 8, alternative reads supporting variants is greater than 2, number of callers is greater than 2 (SomaticSeq call was also assumed to be one of the variant callers), and maximum allele frequency of the corresponding variant in ethnic groups among population databases, 1000 Genomes and gnomAD is less than 0.01.

### Tumor purity-adjusted copy number variation

For copy number variation (CNV) calling, CNVkit program was utilized with each tumor-normal paired BAM files as input. CNVkit created a reference (.cnn) using the matched normal BAM and calculated the copy number from tumor BAM taking into account both on- and off-target reads. As a result, copy ratios (.cnr) file and copy segments (.cns) file were obtained. In order to estimate tumor purity and subpopulations, we run THetA2 using the cns file and the mutect1 somatic variant (.vcf) results. THetA2 estimates the most likely decomposed fraction(s) of clonal and/or subclonal tumor population. We simply added the estimated clonal/subclonal tumor fraction(s) as a represented tumor purity score per sample, then applied it to run CNVkit again (CNVkit’s *call* command) with the cns file and finally obtained tumor purity-adjusted copy number information. Copy number variation burden in a sample was defined as the number of amplified or deleted genes.

### Genomic profile analysis

TMB was reported as mutations/megabases (Mut/Mb). As there is no standard cutoff for TMB to define TMB-high patients with breast cancer, we searched for cutoff values from previous clinical trials of immunotherapy for breast cancer [11]. We calculated the mean value of the TMB cutoffs and estimated the TMB cutoff for TMB-high patients to be 8 muts/Mb. Patients with mismatch repair gene mutations were defined as those with pathogenic somatic or germline mutations in related genes, including *MLH1*, *MLH2*, *MSH3*, *MSH6*, and *MUTYH*.

A list of homologous recombination deficiency (HRD)-related genes was constructed from a gene list from a previous clinical trial that evaluated the efficacy of olaparib for HRD prostate cancer [12] and from several other studies [13–15] describing the genes associated with HRD. HRD-related genes included *BRCA1*, *BRCA2*, *ATM*, *BRIP1*, *BARD1*, *CDK12*, *CHEK1*, *CHEK2*, *FANCA*, *FANCL*, *PALB2*, *PPP2R2A*, *RAD51B*, *RAD51C*, *RAD51D*, *RAD54L*, *FANCD2*, *RAD50*, and* ERCC2.*

### Mutational Signature

We used the Mutalisk R package and COSMIC Mutational Signatures v2 to analyze the mutational signatures of tumor samples [16]. Using annotated and filtered VCF files as inputs, we obtained the decomposed fraction score for 30 mutational signatures per sample and compared it with the samples from the cohort.

### Homologous recombination deficiency

To measure the genomic instability caused by possible defects in DNA repair pathways, we analyzed the HRD score based on whole-exome sequencing (WES) data, which is the sum of three metrics of chromosome-level aberrations: loss of heterozygosity (LOH), telomeric-allelic imbalance (TAI), and large-scale state transitions (LST). Using the segmentation results from the Sequenza tool [17], HRD scores were generated using the scarHRD [18] R package (v0.1.1).

### Transcriptomic profile analysis

PAM50 analysis was conducted as previously described [19], in which normalized expression data were extracted for the 50 genes of interest, and Spearman’s rank correlation values were calculated between samples and each PAM50 subtype centroid. Each sample was classified based on PAM50 classes of the most correlated subtypes.

We used transcripts per million normalized RNA sequencing data for gene ontology (GO) and gene set enrichment analyses. The GO analysis was performed using DAVID GO [20]. Benjamin *p* values were used for the analysis. Gene set enrichment analysis and single-sample gene set enrichment analyses were performed using GSEA version 4.0 [21]. The gene set used for gene set enrichment analysis was the hallmark gene set [22], immune-related signatures from the literature on the immune landscape [23], and the AP gene set from the available literature [8]. To analyze tumor-infiltrating lymphocytes (TIL) in the tumor microenvironment based on RNA expression, we performed CIBERSORT analysis [24], which deconvolutes cell-type proportions from bulk RNA expression data. In principle, the IOBR [25] R package (deconvo_tme function) was used to obtain the relative proportion scores of 22 immune cell types per sample, using normalized fragments per kilobase of transcripts per million mapped read values as input.

### External dataset

We used the genomic and transcriptomic datasets provided in the supplementary materials of a previous study [8], an exploratory biomarker analysis of a clinical trial for eribulin plus pembrolizumab or placebo for HRpos breast cancer. We conducted an identical analysis using an external dataset.

### Statistical analysis

The Wilcoxon rank-sum test was used to compare the values between groups. Fisher’s exact test was used to compare response rates between groups. We used the log-rank test and showed the results using Kaplan–Meier curves to compare survival between groups. The Cox proportional hazards model was used to evaluate the survival hazard ratios of the variables. Statistical significance was set at *p* < 0.05, and the significance tests were not corrected for multiple comparisons owing to the exploratory nature of this study. Statistical analyses of the clinical outcomes were performed using the R software (version 4.0.3).

## Results

### Clinical characteristics

Among the 90 patients included in the trial, WES and whole-transcriptome sequencing (WTS) data were acquired from 76 and 58 patients, respectively. In each WES and WTS cohort, the clinical characteristics and efficacies were comparable to those of the entire trial patient cohort (Table 1). In addition, neither PD-L1 nor TIL were associated with clinical outcomes in each cohort in terms of the PFS6 rate and PFS (Supplementary Figure 1).

**Table 1:** Patient characteristics

	WES cohort (*n* = 76)	WTS cohort (*n* = 58)
Median age (range)	52.5 (31–71)	53 (31–71)
Subtype		
HRpos	40 (52.6%)	29 (50.0%)
TNBC	36 (47.4%)	29 (50.0%)
Prior Line of Tx		
None	15 (19.7%)	10 (17.2%)
1	38 (50.0%)	29 (50.0%)
2	23 (30.3%)	19 (32.8%)
Prior cyclin-dependent kinase 4/6 inhibitor		
Yes	17 (22.4%)	14 (24.1%)
No	59 (77.6%)	44 (75.9%)
Prior anthracycline		
Yes	50 (65.8%)	44 (75.9%)
No	26 (34.2%)	14 (24.1%)
Prior taxanes		
Yes	72 (94.7%)	56 (96.6%)
No	4 (5.3%)	2 (3.4%)
PFS6		
Responder	28 (36.8%)	20 (34.5%)
Nonresponder	48 (63.2%)	38 (65.5%)
PD-L1 (SP142)		
Positive	16 (21.1%)	15 (25.9%)
Negative	60 (78.9%)	43 (74.1%)

*HRpos* hormone-positive breast cancer, *PD-L1* programmed cell death ligand 1, *PFS6* 6-month progression-free survival, *TNBC* triple-negative breast cancer, *Tx* treatment, *WES* whole-exome sequencing, *WTS* whole transcriptomic sequencing

### Genomic profiles and outcomes

In total, 268 oncogenic somatic mutations were detected in the WES cohort (Supplementary Table 1). The most common genetic alterations were in *TP53* (55 mutations), followed by *GNAQ* (22 mutations), *PIK3CA* (20 mutations), *KMT2C* (13 mutations), and *ESR1* (10 mutations). Only one patient had a germline *BRCA2* truncating mutation; no patient had a germline *BRCA1* mutation.

**Fig. 1:**
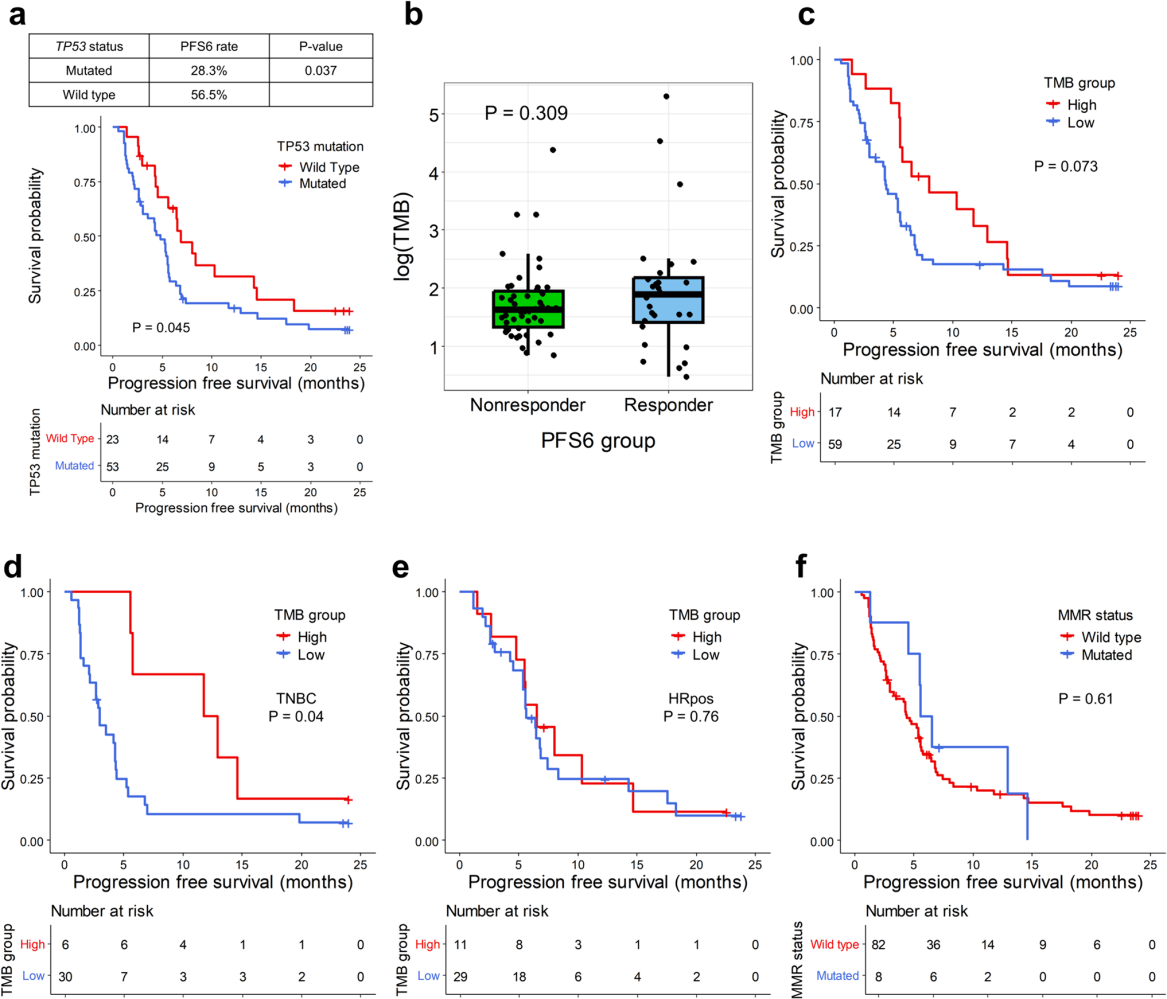
Tumor mutation burden and efficacy. **a** Kaplan–Meier curves showing the progression-free survival (PFS) according to *TP53* mutation status. The red line represents the mutant group, and the blue line denotes the wild-type group. Censored data are marked with vertical segments. The risk table is shown below the curves; the log-rank *p* value is shown. The right table presents the PFS6 rate according to *TP53* mutation status. **b** Box plots illustrate the log value of tumor mutation burden (TMB) according to the PFS6 group; each dot represents a single patient. The *p* value is shown. **c** Kaplan–Meier curves showing the PFS according to TMB-high or TMB-low status. The red line represents the TMB-high group, and the blue line indicates the TMB-low group. **d**, **e** The Kaplan–Meier curves show the PFS according to TMB-high or TMB-low among TNBC and HRpos patients, respectively. **f** The Kaplan–Meier curves show the PFS according to MMR status. Censored data are marked with vertical segments. The risk table is shown below the curves; the log-rank *p* value is shown. Patients who achieved PFS ≥ 6 months were defined as PFS6-responders and otherwise as PFS6-nonresponders

Patients with a *TP53* mutation showed significantly poor PFS6 rates (28.3% vs. 56.5%, *p* = 0.037) and PFS (median PFS 6.9 months [95% confidence interval (CI) 5.6–14.6] vs. 4.8 months [95% CI 3.0–5.6], *p* = 0.045, Fig. 1a). No other single gene mutations with a frequency of more than three were associated with the PFS6 rate or PFS (Supplementary Table 2).

#### TMB was associated with favorable outcomes

The median TMB in the WES cohort was 5.3 mut/Mb (ranging from 1.6–200.3). The median TMB by subtypes was 6.0 mut/Mb (range 1.9–93.5) for HRpos breast cancer and 4.7 mut/Mb (range 1.6–200.3) for TNBC. PFS6-responders show no significantly different TMB values compared with PFS6-nonresponders (median TMB 6.6 vs. 5.1 mut/Mb, *p* = 0.309, Fig. 1b). By using a TMB cutoff of 8 Mut/Mb, TMB-high patients showed higher PFS compared with TMB-low patients without statistical significance (median PFS 8.0 months, 95% CI 5.6–14.6 for TMB-high and 4.3 months, 95% CI 3.0–5.6 for TMB-low, *p* = 0.07, Fig. 1c), with long responders in both TMB-high and TMB-low groups. The tendency of PFS difference was observed in TNBC but not HRpos (Fig. 1d, e).

Eight patients harbored mutations in mismatch repair genes (MMR deficient patients), five had somatic mutations, and three had germline mutations. Although MMR deficient patients tended to have higher PFS6 rates (50.0% vs. 31.7%, *p* = 0.433) and PFS compared with other patients, the differences were not statistically significant (median PFS 6.0 months, 95% CI 5.5–not available [NA] vs. 4.4 months, 95% CI 3.0–5.6, *p* = 0.61; Fig. 1f).

#### HRD tended to show poor outcomes

**Fig. 2:**
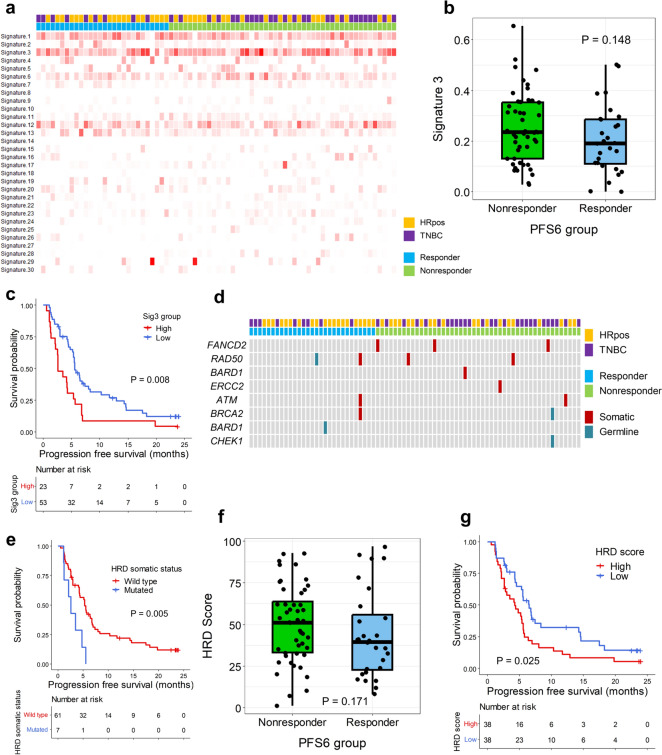
Homologous recombination deficiency and efficacy. **a** Heatmap showing the signature proportions in each patient. Each column represents a patient, and each row represents a mutational signature. On the upper side, each patient is marked according to the tumor subtype and the PFS6 group as the legend. In the heatmap, the degree of redness correlates with the proportion of the signature. **b** Box plots showing the proportion of signature 3 according to the PFS6 group. Each dot represents a patient. The *p* value is shown. **c** Kaplan–Meier curves showing the progression-free survival (PFS) according to high signature 3 or low signature 3. The red line represents the high signature 3 group, and the blue line denotes the low signature 3 group. Censored data are marked with vertical segments. The risk table is shown below the curves; the log-rank *p* value is shown. **d** Landscape plot showing the HRD-related gene mutation status in patients. Each column represents a patient, and each row represents a gene. On the upper side, each patient is marked according to the tumor subtype and the PFS6 group as the legend. The columns are ordered left to right by PFS from the longest to the shortest. The red marks indicate patients harboring somatic gene mutations, while the grey marks indicate patients harboring germline gene mutations. **e** Kaplan–Meier curves show the progression-free survival (PFS) according to HRD-related gene mutation status. The red line represents patients with a somatic mutation, and the blue line represents those without a somatic mutation in the HRD-related genes. Censored data are marked with vertical segments. The risk table is shown below the curves. The log-rank *p* value is shown. **f** Box plots showing the HRD score according to the PFS6 group. Each dot represents a patient; the *p* value is shown. **g** Kaplan–Meier curves showing the PFS according to HRD scores. The red line represents the high HRD score group, and the blue line indicates the low HRD score group. Censored data are marked with vertical segments. The risk table is shown below the curves; the log-rank *p* value is shown. Patients who achieved PFS ≥ 6 months were defined as PFS6-responders and otherwise as PFS6-nonresponders

As HRD is an important feature of breast cancer and susceptibility to DNA-damaging agents in breast cancer is common [26], we analyzed whether there was any association between mutational signatures and the efficacy of eribulin and nivolumab. We found that signature 3 (Sig3), which is related to HRD tumors [27], was frequently observed in our cohort (Fig. 2a). PFS6-nonresponders tended to have higher Sig3 proportions compared with PFS6-responders without statistical significance (*p* = 0.148, Fig. 2b). This tendency was observed in HRpos but not in TNBC (*p* = 0.163 for HRpos and *p* = 0.927 for TNBC; Supplementary Figure 2a). The PFS analysis performed after selecting patients with the top 30% Sig3 proportion values showed significantly shorter PFS for the high signature 3 group (median PFS 2.6 months, 95% CI 2.2–5.6 vs. 5.6 months, 95% CI 5.3–8.3, *p* = 0.008, Fig. 2c). PFS differences were observed in both HRpos and TNBC (Supplementary Figure 2b).

We investigated the HRD and outcomes by dividing the patients according to their HRD-related gene mutation status (Fig. 2d). When MMR deficient patients were excluded, patients with somatic HRD-related gene mutations showed significantly lower PFS6 rates than other patients (Table 2). In addition, patients harboring somatic HRD-related gene mutations showed significantly shorter PFS compared with other patients (median PFS 3.5 months, 95% CI 2.2–NA vs. 5.5 months [95% CI 4.3–6.9], *p* = 0.02; Supplementary Figure 2c); these results were more evident after excluding MMR deficient patients (median PFS 2.6 months, 95% CI 1.2–NA vs. 5.4 months, 95% CI 4.3–6.8, *p* = 0.005, Fig. 2e). This tendency was significant in HRpos and not in TNBC (*p* = 0.007 for HRpos and *p* = 0.18 for TNBC; Supplementary Figure 2d).

**Table 2:** PFS6 rate according to HRD-related gene mutation status

HRD-gene mutation status	PFS6 rate	*p* value
All patients		
Somatic mutation		0.142
Yes	11.1% (1/9)	
No	40.3% (27/67)	
Germline mutation		0.551
Yes	66.7% (2/3)	
No	35.6% (27/73)	
Somatic/germline mutation		0.518
Yes	25.0% (3/12)	
No	39.1% (25/64)	
Excluding MMR defect patients		
Somatic mutation		0.046
Yes	0% (0/7)	
No	39.3% (24/61)	
Germline mutation		1.0
Yes	50.0% (1/2)	
No	34.8% (23/66)	
Somatic/germline mutation		0.144
Yes	11.1% (1/9)	
No	39.0% (23/59)	

Next, we investigated the HRD score, which represents the degree of DNA damage caused by HRD. PFS6-nonresponders tended to have higher HRD scores than PFS6-responders without statistical significance (median HRD score: 51 vs. 39.5, *p* = 0.171; Fig. 2f). When patients were divided into two groups by median HRD score, which was 44, patients with high HRD scores showed lower PFS6 rates (23.7% vs. 50.0%, *p* = 0.031) and significantly shorter PFS compared to patients with low HRD scores (median PFS 4.2 months, 95% CI 2.6–5.6 vs. 6.5 months, 95% CI 5.3–14.3], *p* = 0.025, Fig. 2g). This tendency was also observed when we analyzed patients by subtype without statistical significance (*p* = 0.12 for HRpos and *p* = 0.37 for TNBC; Supplementary Figure 2e).

We applied the same gene list to a previously reported external genomic dataset from a clinical trial of eribulin plus pembrolizumab for HRpos by Keenan et al. [8] and observed that patients with and without HRD-related gene mutations showed similar PFS6 rates (38.5% vs. 35.7%, *p* = 1.0) and PFS (*p* = 0.33; Supplementary Figure 2f).

#### Mutational signature 25 was associated with poor clinical outcomes

To determine whether other mutational signatures were associated with clinical outcomes, we compared other mutational signatures between PFS6-responders and PFS6-nonresponders. Among the signatures, signature 25 (Sig25) was more frequently observed in PFS6-nonresponders (22.9% in PFS6-nonresponders and 3.6% in PFS6-responders, *p* = 0.047).

**Fig. 3:**
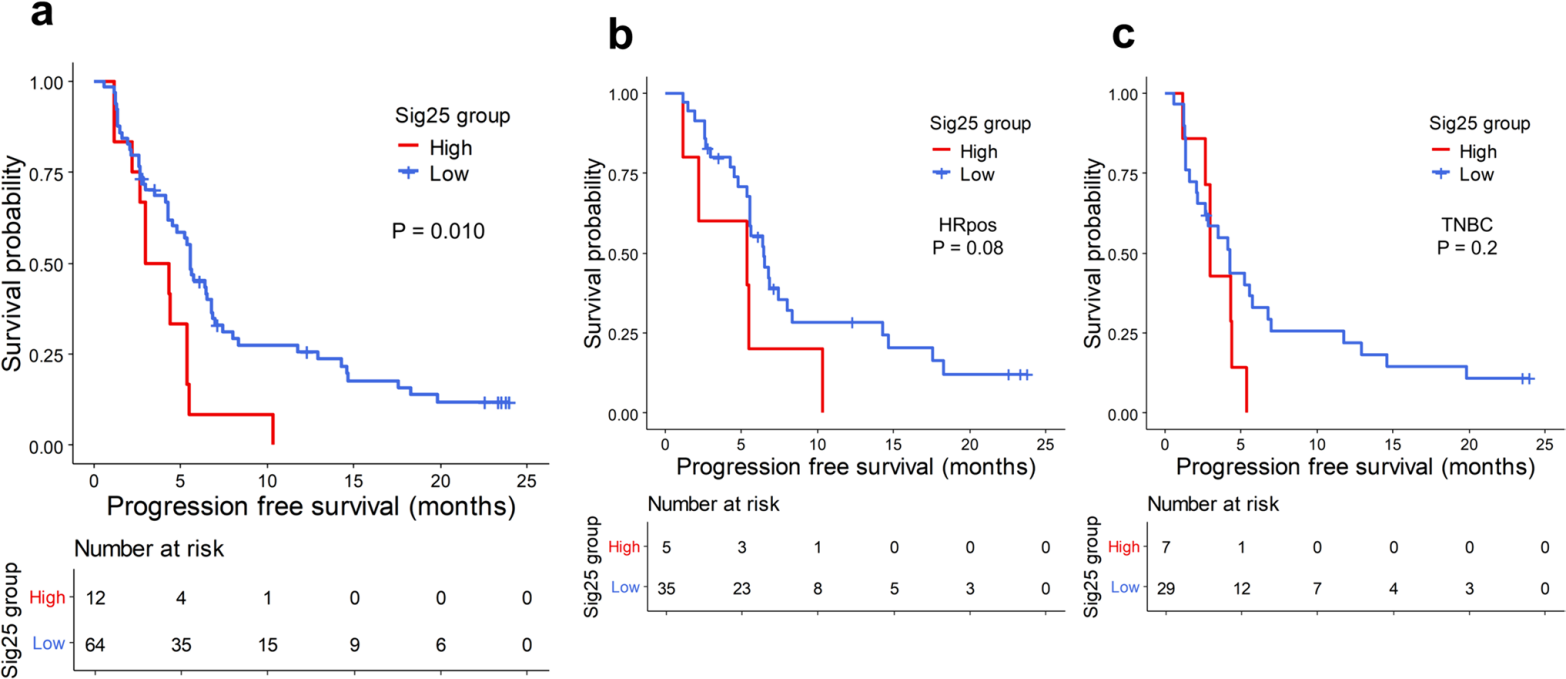
Mutational signature 25 and PFS. **a** Kaplan–Meier curves showing PFS according to Sig25 in the whole WES cohort and **b** HRpos and **c** TNBC, respectively. The red line represents the patients with signature 25, and the blue line denotes the patients without signature 25 group. Censored data are marked with vertical segments. The risk table is shown below the curves; the log-rank *p* value is shown

The Sig25 pattern, which was lacking in 64 patients, was identified in 12 patients, among whom 9 had prior systemic treatments. Patients with Sig25 showed significantly shorter PFS compared with patients without it (median PFS 3.7 months, 95% CI 2.6–NA vs. 5.6 months, 95% CI 4.5–6.9, *p* = 0.01; Fig. 3a). This tendency was observed in both subtypes (*p* = 0.08 for HRpos and *p* = 0.2 for TNBC; Fig. 3b, c).

#### Copy number variations were not associated with clinical outcomes

The copy number variation (CNV) burden did not significantly differ between PFS6-responders and PFS6-nonresponders (*p* = 0.629; Supplementary Figure 3a); this was also consistent when the CNV burden was adjusted for tumor purity, although PFS6-responders tended to have a higher adjusted CNV burden (*p* = 0.330; Supplementary Figure 3b). When patients were divided into two groups by median CNV burden value, there was no significant PFS difference between groups (median PFS 5.6 months, 95% CI 4.8–10.3 for CNV burden high vs. 4.2 months, 95% CI 3.0–6.4 for CNV burden low, *p* = 0.29; Supplementary Figure 3c).

### Transcriptomic profiles and outcomes

#### Breast *cancer* expression subtypes showed a nonsignificant association with clinical outcomes

**Fig. 4:**
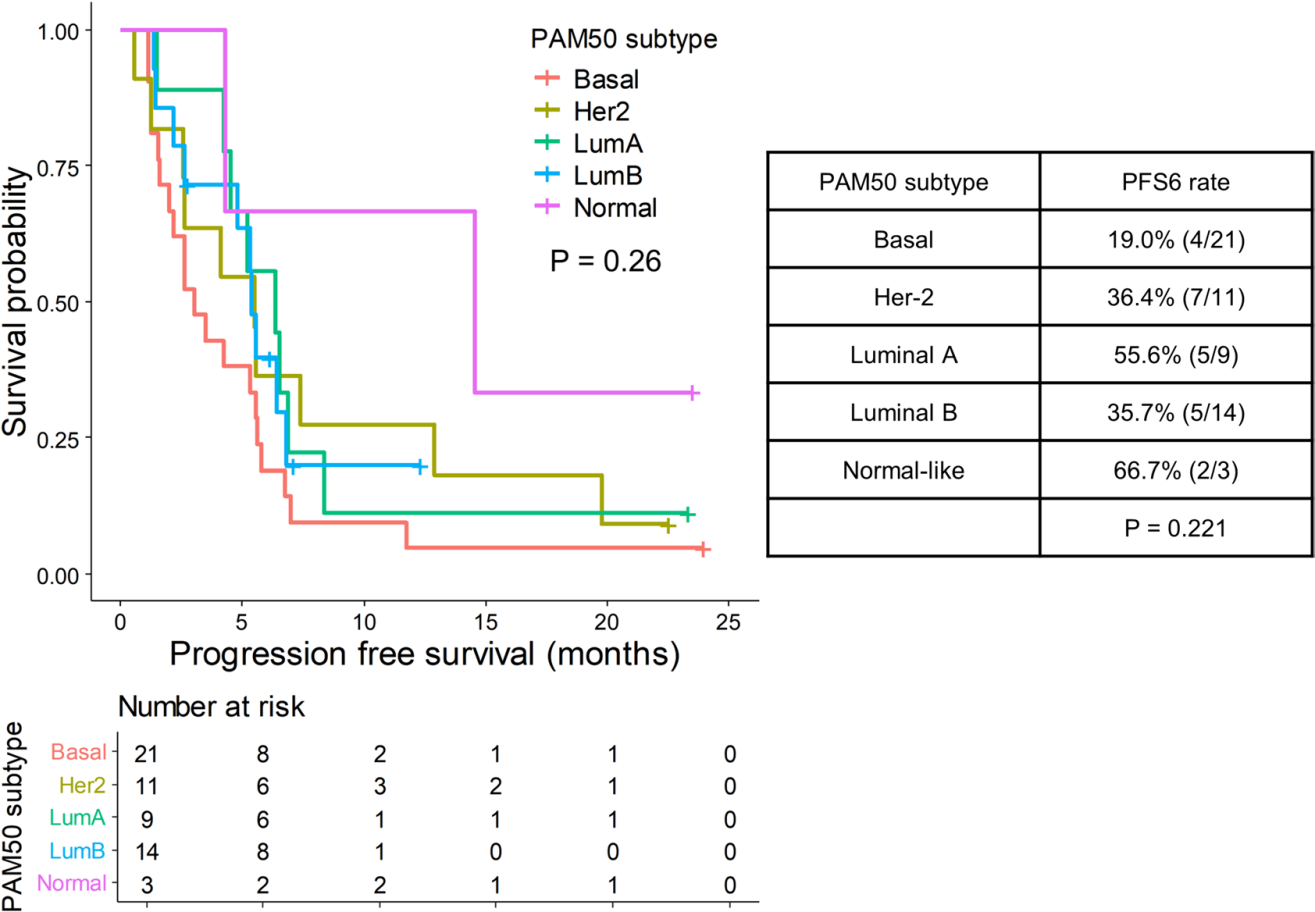
PAM50 subtype and efficacy. On the left, Kaplan–Meier curves for PFS according to PAM50 subtypes are shown. Each color represents each PAM50 subtype as annotated in the legend. Censored data are marked with vertical segments. The risk table is shown below the curves; the log-rank *p* value is shown. On the right, a table summarizing PFS6 rates according to PAM50 subtypes are shown, with *p* values by Fisher’s exact test in the bottom

When we divided the breast cancer expression subtypes (PAM50 subtype) according to expression features [28], we identified nine luminal A, 14 luminal B, 21 basal, 11 HER-2, and three normal-like samples. The normal-like subtype had the highest PFS6 rate (66.7%), whereas the basal subtype had the lowest (19.0%). The PFS results showed similar trends to the PFS6 rate results, although differences between subtypes were not statistically significant (*p* = 0.26; Fig. 4).

#### Cell cycle-related signatures were associated with poor outcomes

**Fig. 5:**
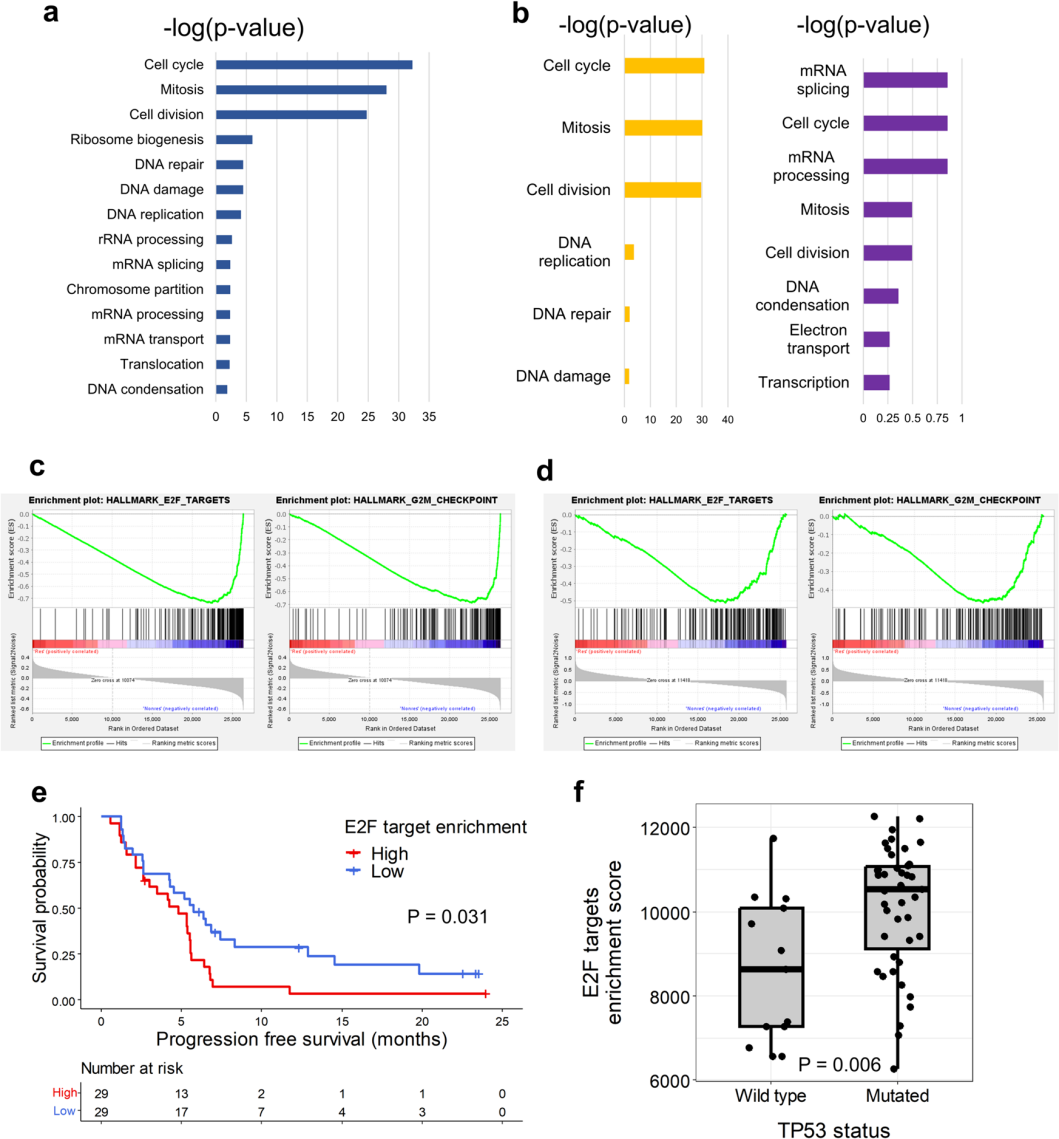
Transcriptomic comparisons between PFS6-responders and PFS6-nonresponders. **a** Enriched gene ontology terms in PFS6-nonresponders. **b** Enriched gene ontology terms in PFS6-nonresponders of HRpos (yellow) and TNBC (purple), respectively. **c** Gene set enrichment analysis results comparing PFS6-responders and PFS6-nonresponders in the whole WTS cohort, showing E2F target and G2M checkpoint pathways being significantly enriched in PFS6-nonresponders. **d** Gene set enrichment analysis results comparing PFS6-responders and PFS6-nonresponders in the external dataset, showing E2F target and G2M checkpoint pathways being significantly enriched in PFS6-nonresponders. **e** Kaplan–Meier curves show the progression-free survival (PFS) according to E2F target enrichment scores. The red line represents the high E2F target enrichment score group, and the blue line denotes the low E2F target enrichment score group. The censored data are marked with vertical segments. The risk table is shown below the curves; the log-rank *p* value is shown. Patients who achieved PFS ≥ 6 months were defined as PFS6-responders and otherwise as PFS6-nonresponders. **f** Boxplot showing E2F targets enrichment scores according to *TP53* mutation status. Each dot represents a patient. The *p* value is shown

We observed 336 and 788 upregulated genes in PFS6-responders and PFS6-nonresponders, respectively. Using GO analysis, we found that the term most enriched by genes upregulated in PFS6-responders was angiogenesis; however, it was not significant according to the Benjamin *p* value (*p* = 0.12). Genes upregulated in PFS6-nonresponders were enriched in biological terms involved in the cell cycle and DNA repair or damage (Fig. 5a). Among these terms, cell cycle and mitotic biological processes were consistently enriched in PFS6-nonresponders when the analyses were performed within each subtype (Fig. 5b).

We then used gene set enrichment analysis to determine whether cell cycle pathways were significantly enriched in PFS6-nonresponders. Hallmark E2F targets and G2M checkpoint pathways were consistently enriched in PFS6-nonresponders among the whole cohort, HRpos, and TNBC patients (Fig. 5c; Supplementary Figure 4a, b). When gene set enrichment analysis using the external dataset was performed after dividing patients according to the PFS6 status, we also observed a tendency to enrich hallmark E2F targets and G2M checkpoint pathways in PFS6-nonresponders (Fig. 5d). We estimated the enrichment score of the E2F pathway signature in each cohort sample using single-sample gene set enrichment analysis. We found that patients with high E2F pathway signature scores (≥ median value) showed significantly shorter PFS compared to patients with low E2F pathway signature scores (median PFS 4.8 months, 95% CI 2.6–5.6 vs. 5.8 months, 95% CI 4.3–12.9, *p* = 0.03, Fig. 5e). These trends were consistent when each subtype was analyzed (Supplementary Figure 4c, d). These results seemed to be associated with *TP53* mutations, as patients harboring *TP53* mutations had significantly higher E2F target enrichment scores (*p* = 0.006; Fig. 5f).

#### B-cell infiltration and AP gene set enrichment were associated with favorable outcomes

**Fig. 6:**
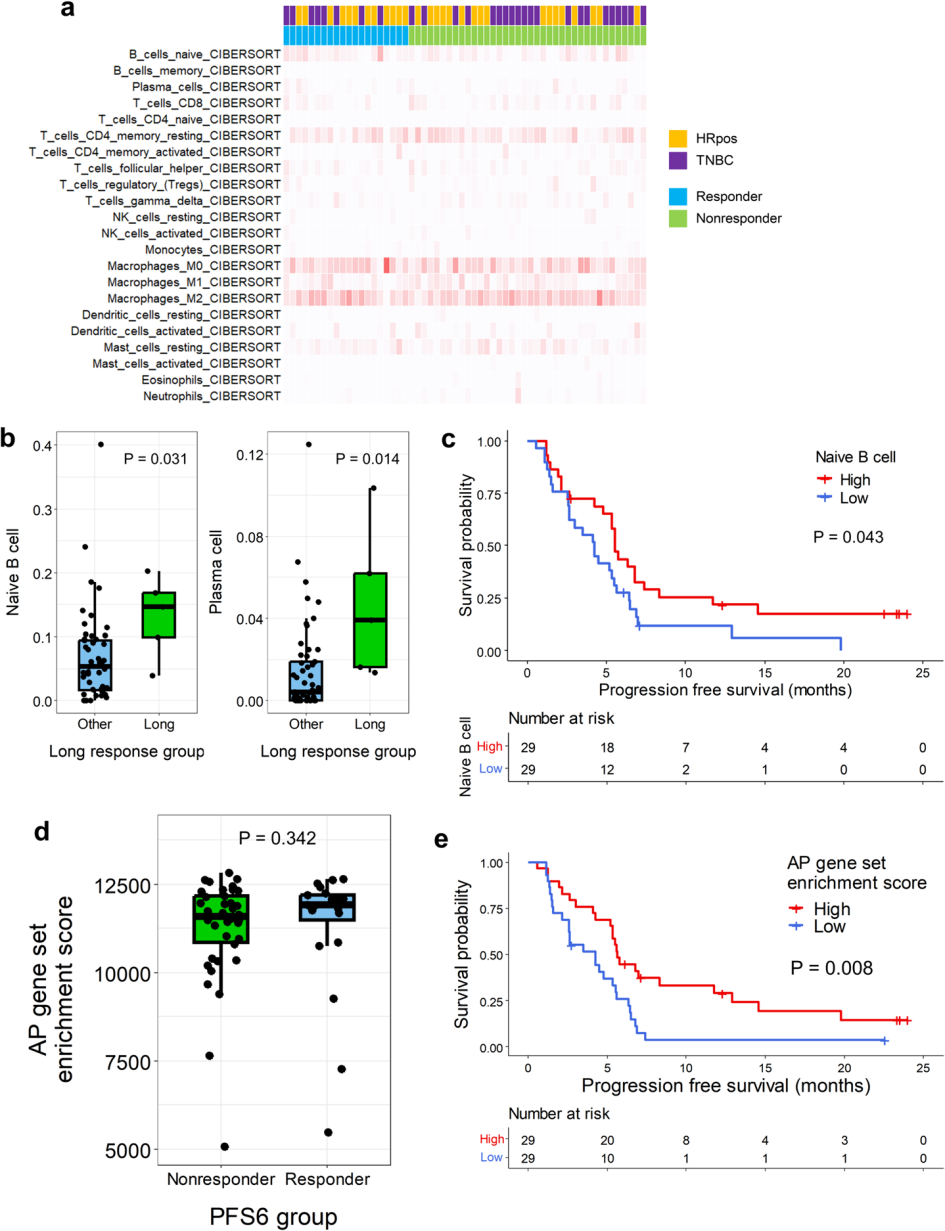
Immune-related gene signatures and the outcome. **a** Heatmap shows the estimation of abundance of each cell types by CIBERSORT analysis. Each column represents each patient, and each row represents each cell type. In the upper side, each patient is marked according to the tumor subtype and the PFS6 group as the legend. In the heatmap, the degree of redness correlates the estimation. **b** Box plots showing the naïve B-cell and plasma cell infiltration proportions according to the long response groups. Each dot represents each patient. The *p* value is shown. **c** The Kaplan–Meier curves show progression-free survival (PFS) according to naïve B-cell infiltration. The red line represents the high naïve B-cell infiltration group, and the blue line denotes the low naïve B-cell infiltration group. Censored data are marked with vertical segments. The risk table is shown below the curves; the log-rank *p* value is shown. **d** The box plots show the antigen presentation (AP) gene set enrichment scores according to the PFS6 group. Each dot represents a patient; the *p* value is shown. **e** Kaplan–Meier curves show the PFS according to AP gene set enrichment scores. The red line represents the high AP gene set enrichment score group, and the blue line indicates the low AP gene set enrichment score group. The censored data are marked with vertical segments. The risk table is shown below the curves; the log-rank *p* value is shown. Patients who achieved PFS ≥ 6 months were defined as PFS6-responders and otherwise as PFS6-nonresponders

As immune-related genes are often highly expressed in tumors from immunotherapy responders, we examined immune-related signatures to determine whether any of these signatures were significantly associated with efficacy. However, major immune-related expression signatures from previous literature [23] did not differ between PFS6-responders and PFS6-nonresponders (Supplementary Figure 5a). None of the TIL subsets significantly differed between PFS6-responders and PFS6-nonresponders (Fig. 6a, Supplementary Figure 5b). As a proportion of PFS6-responders might have responded to eribulin but not to nivolumab, we further defined long responders (PFS ≥ 18 months) and compared the immune-related signatures and TIL profiles of such patients with those of other patients. Although none of the immune-related signatures significantly differed between long responders and other patients, the proportions of naïve B-cells and plasma cells were significantly higher in tumors from long responders (Fig. 6b, Supplementary Figure 5c). Among these two cell subtypes, patients with a higher proportion of naïve B-cells (≥ median value) showed significantly longer PFS (median PFS 5.6 months [95% CI 4.0–8.3] vs. 4.2 months [95% CI 2.6–5.6], *p* = 0.043, Fig. 6c).

Naïve B-cells are involved in the AP process, and we hypothesize that this result aligns with a previous study that suggested that upregulated AP gene set expression was associated with the response to eribulin plus immunotherapy [8]. AP gene set enrichment scores were high in PFS6-responders, although the differences were not statistically significant (Fig. 6d). In addition, when the patients were divided according to the AP gene set enrichment scores, patients with high scores (≥ median value) showed significantly longer PFS compared with other patients (median PFS 5.6 months [95% CI 5.3–12.9] vs. 4.2 months [95% CI 2.6–5.6], *p* = 0.008, Fig. 6e).

### Multivariate analysis of molecular profiles associated with clinical outcomes

We performed a multivariate Cox proportional hazard analysis of patients with DNA and RNA data, using potential biomarkers associated with PFS in patients who received eribulin plus nivolumab (Table 3). In the multivariate analysis, high TMB (hazard ratio HR 0.45, 95% CI 0.21–0.94, *p* = 0.033) and high AP gene set enrichment scores (hazard ratio 0.46, 95% CI 0.23–0.93, *p* = 0.031) were associated with longer PFS, while somatic HRD-related gene mutations were associated with shorter PFS (hazard ratio 2.62, 95% CI 1.08–6.40, *p* = 0.034).

**Table 3:** Cox proportional hazard analysis of biomarkers for PFS

Feature	Univariate analysis		Multivariate analysis	
	HR (95% CI)	*p* value	HR (95% CI)	*p* value
Subtype				
HRpos	Reference		Reference	
TNBC	1.26 (0.72–2.20)	0.427	1.72 (0.88–3.36)	0.114
TMB group				
Low	Reference		Reference	
High	0.52 (0.26–1.03)	0.061	0.45 (0.21–0.94)	0.033
Somatic HRD-related gene status				
Wild type	Reference		Reference	
Mutated	2.52 (1.15–5.53)	0.022	2.62 (1.08–6.40)	0.034
Signature 25				
Low	Reference		Reference	
High	2.64 (1.13–6.19)	0.025	2.01 (0.83–4.90)	0.124
E2F target enrichment score				
Low	Reference		Reference	
High	1.87 (1.05–3.31)	0.033	1.43 (0.74–2.74)	0.284
AP gene set enrichment score				
Low	Reference		Reference	
High	0.46 (0.26–0.83)	0.009	0.46 (0.23–0.93)	0.031

*CI* confidence interval, *HR* hazard ratio

## Discussion

Here, we described the genomic and transcriptomic profiles related to the efficacy of eribulin plus nivolumab in patients with HER-2-negative breast cancer from pretreatment tumor tissues. Our results suggest that high TMB and AP gene set enrichment is associated with good efficacy, whereas HRD, Sig25, and high E2F signature are associated with poor efficacy. Although the eribulin plus immunotherapy combination regimen requires further evaluation for use in real-world clinical practice, the associations identified herein may have implications for the use of immunotherapy combination in these tumors.

TMB and MMR defects are closely related to each other, and both are related to immunotherapy in various types of cancer [29, 30]. TMB is associated with the good efficacy of eribulin plus immunotherapy [8], and similar results were observed in our trial cohort. However, the exact cutoff for high TMB in breast cancer remains to be elucidated [11]. Relatively low TMB in breast cancer compared to other immunogenic cancers suggests that a lower TMB cutoff may be appropriate, as in our study [31].

HRD causes impaired DNA repair and aberrant DNA fragmentation, contributing to increased neoantigens and activating innate immunity via the cGAS-STING pathway [32]. Therefore, it was hypothesized that HRD might serve as an immunotherapy biomarker. However, a recent TCGA analysis showed that most cancer types displaying HRD had immunologically cold tumors [33]. A clinical trial of immunotherapy for ovarian cancer showed an objective response rate of only 6–22%, regardless of HRD status [34]. These results imply that the use of HRD as a biomarker for immunogenicity is complicated. Although the detailed mechanism remains elusive, one of the hypotheses for HRD not acting as immunogenic is that DNA changes caused by HRD, which consist of LOH, TAI, and LST, are larger-scale DNA changes compared to single nucleotide variants and mismatches [18]. Therefore, DNA changes induced by HRD may act similarly to the CNV burden, which inversely correlates with immunogenicity [35]. Furthermore, the chronic activation of the cGAS-STING pathway can induce an immunosuppressive tumor microenvironment [36]. Therefore, using the HRD status as a biomarker for clinical trials using immunotherapy requires caution, as the status seems more complex than that of TMB.

We found that Sig25 expression was associated with poor efficacy. Sig25 was first detected in Hodgkin’s lymphoma cell lines from patients who underwent chemotherapy [27, 37]. As both Hodgkin’s lymphoma and breast cancer treatments often involve anthracyclines, which are DNA-intercalating agents, the signature may be associated with DNA damage caused by anthracyclines. Most patients with Sig25 in our cohort had received prior systemic treatment. However, little is known about Sig25 expression. Therefore, further studies on the features related to Sig25 generation and their effect on the efficacy of the combination regimen are required.

The clinical relevance of *TP53* mutations in breast cancer is not well established [38]. Nevertheless, *TP53* mutations were associated with cell cycle-related signatures, and both were associated with poor efficacy in our study. While it is unclear whether an enhanced cell cycle is associated with resistance to immune checkpoint inhibitors [39, 40] based on our study, further studies focused on the development of biomarkers for the cell cycle, especially the E2F target gene, may provide additional information on resistance to chemotherapy and immunotherapy in patients with breast cancer.

We found that B-cell infiltration and enrichment of the AP gene set were associated with favorable outcomes, consistently with the biomarker analysis of eribulin plus pembrolizumab for HRpos [8]. Nonetheless, none of the T-cell subtype infiltrations or interferon-related signatures were associated with the outcomes. These results support the importance of MHC-II in BC immunotherapy of breast cancer [8]. In a previous study, highly naïve B-cell and memory B-cell signatures based on single-cell RNA sequencing of tumor-infiltrated B cells in breast cancers showed significantly prolonged survival [41]. Even though the relationships between TIL, tumor microenvironment, and tumor cells are extremely complex, pretreatment tumor biopsy samples could not represent all the features of the heterogeneous immune tumor microenvironment [42]. Thus, further studies with single-cell and spatial transcriptomics are warranted to dissect the immune microenvironment and determine biomarkers for the combination regimen. In addition, temporal changes in the tumor microenvironment after immunotherapy administration could be effective biomarkers for combination regimens. We collected temporal blood samples to evaluate biomarkers such as T cells and cytokines and intend to conduct further investigations.

The findings from genomic and transcriptomic profiles suggest that multiple factors influence treatment outcomes, rather than a single biomarker. Therefore, it is important to consider the complex associations and interactions between biomarkers and treatment outcomes. However, in real-world clinical practice, such comprehensive analyses are often not feasible, and obtaining sufficient biopsy samples of tumor tissues can be challenging. Further studies should focus on determining what to evaluate among the biomarkers for predicting the response to combination treatments, taking into account our study results. Additionally, noninvasive biomarkers, such as nuclear medicine imaging to target tumor microenvironment components, may be utilized based on our findings on expression profiles [43, 44].

This study has some limitations. First, this study is an exploratory analysis with a largely descriptive nature. Findings in our study that contrast with previous knowledge must be interpreted carefully, especially those from the HRD analysis, as there are likely many hidden variables affecting how HRD status influences the efficacy of the combination regimen. Second, the use of a combination regimen and the lack of a control arm make it difficult to unequivocally identify which effects are attributable to the treatments. The combination with chemotherapy could have either augmented or diminished the effects of immunotherapy and vice versa. However, as combination regimens are becoming more prevalent when using immunotherapy, studies evaluating the biomarkers for the combination regimen are necessary to effectively measure the combined clinical and biological effects of each medication. Third, the tissues used in this study were obtained from stored tumor FFPE tissues, not fresh ones. Nucleic acids, especially RNAs, could have been degraded in the FFPE tissues; therefore, there could have been biases. Further analysis of fresh tissues is required to determine the biomarkers for combination regimens. Fourth, there was a lack of sufficient validation using external datasets. Therefore, it is necessary to confirm our findings in other cohorts.

In conclusion, we presented the genomic and transcriptomic profiles linked to the combination of eribulin and nivolumab, along with several points requiring further investigation and clarification. Specifically, TMB and AP gene set enrichment were associated with favorable efficacies while HRD, Sig25, and cell cycle related signatures were associated with poor efficacies. Our study underscores the complexity of using biomarkers like HRD, and the importance of cell cycle and B-cell-related markers. The need for further research, particularly with additional cohorts, is emphasized to validate these preliminary findings and enhance the understanding of combination treatment in HER-2-negative breast cancer.

## Supplementary Information

Below is the link to the electronic supplementary material.

Supplementary file1 (PDF 1347 kb)

## Data Availability

Raw data for this study were generated at PentaMedix Incorporation. The raw data of sequencing data in this study are available at BioProject database (BioProject PRJNA1099131).

## Abbreviations

AP: Antigen presentation
CD: Combinatorial dual
CNV: Copy number variation
CI: Confidence interval
FFPE: Formalin-fixed: paraffin-embedded
FPKM: Fragments per kilobase of transcript per million mapped read
GO: Gene ontology
GSEA: Gene set enrichment analysis
HRD: Homologous recombination deficiency
HRpos: Hormone-positive breast cancer
HER-2: Human epidermal growth factor receptor-2
LST: Large-scale state transitions
LOH: Loss of heterozygosity
MBC: Metastatic breast cancer
MMR: Mismatch repair
Mut/Mb: Mutations/megabases
NGS: Next-generation sequencing
PD-L1: Programmed cell death ligand-1
PFS: Progression-free survival
PFS6: 6-Month progression-free survival
RPK: Reads per kilobase
TMB: Tumor mutation burden
TNBC: Triple-negative breast cancer
TPM: Transcripts per million
TIL: Tumor-infiltrating lymphocytes
TAI: Telomeric-allelic imbalance
Sig25: Signature 25
UDI: Unique dual indexed
WES: Whole-exome sequencing
WTS: Whole-transcriptome sequencing
