# Supplementary material for: Genomic and transcriptomic profiles associated with response to eribulin and nivolumab combination in HER-2-negative metastatic breast cancer
Source: Cancer Immunol Immunother. 2024 Aug 6;73(10):197. doi: 10.1007/s00262-024-03782-7 (PMC11303363; doi:10.1007/s00262-024-03782-7)
Supplement: Supplementary file 1 — Supplementary file1 (PDF 1347 kb) [file 262_2024_3782_MOESM1_ESM.pdf]

SUPPLEMENTARY FIGURES

Supplementary Figure 1

a

PD-L1 Group	PFS6 rate	P-value
Positive	31.3%	0.773
Negative	38.3%	

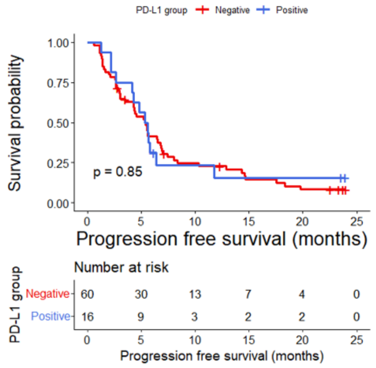

b

PD-L1 Group	PFS6 rate	P-value
Positive	26.7%	0.541
Negative	73.3%	

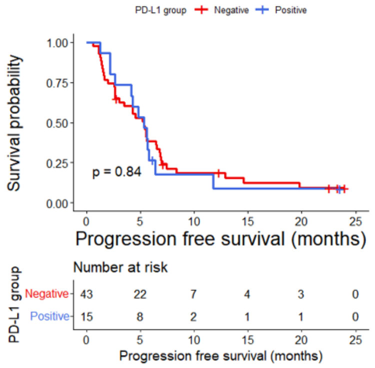

c

TIL Group	PFS6 rate	P-value
High	33.3%	0.643
Low	33.3%	
Negative	44.4%	

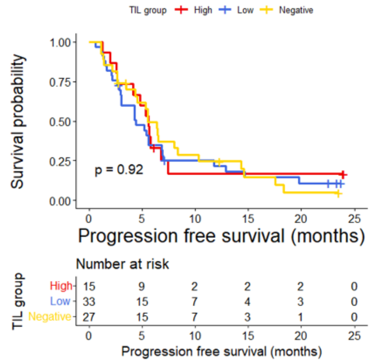

d

TIL Group	PFS6 rate	P-value
High	30.8%	1.0
Low	34.6%	
Negative	36.8%	

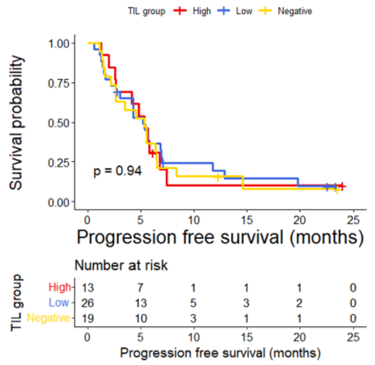

Supplementary Figure 1. Patient outcomes according to PD-L1 and TIL status

The upper tables show PFS6 rates, and the lower Kaplan-Meier curves show PFS. (a) WES cohort divided according to PD-L1 status (b) WTS cohort divided according to PD-L1 status. (c) WES cohort divided according to TIL status. (d) WTS cohort divided according to TIL status.

Supplementary Figure 2

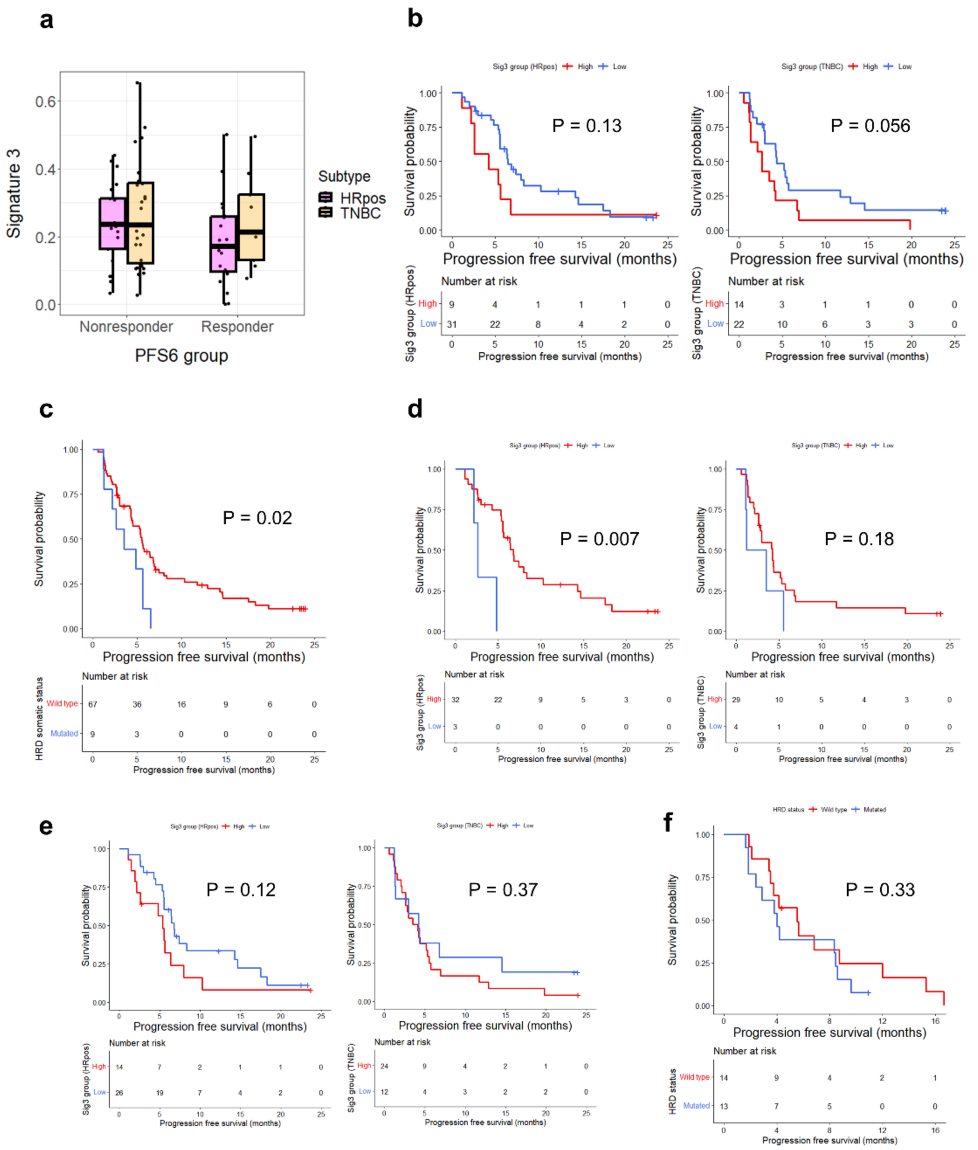

Supplementary Figure 2. Outcomes according to HRD

(a) The boxplots show the proportion of signature 3 according to PFS6 group in each subtype. (b) Kaplan-Meier curves showing PFS according to Sig3 in Hrpos and TNBC, respectively. (c) PFS according to somatic HRD mutation status in whole WES cohort. (d) PFS according to somatic HRD mutation status after excluding MMR defect patients in each subtype. (e) PFS according to HRD scores in each subtype (HRpos on the left, TNBC on the right). (f) PFS according to somatic HRD mutation status in external dataset.

Supplementary Figure 3

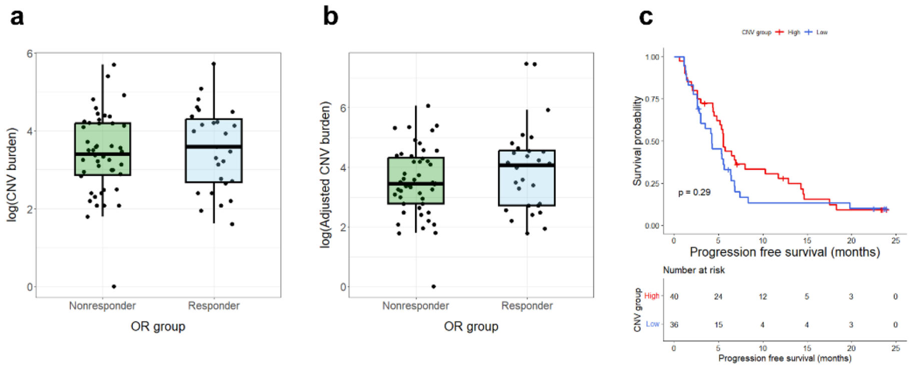

Supplementary Figure 3. Outcomes according to CNV burden

(a) The boxplots show the log values of CNV burden according to PFS6 group. (b) The boxplots show the log values of adjusted CNV burden according to PFS6 group. (c) Kaplan-Meier curves showing PFS according to Sig25 in the whole WES cohort.

Supplementary Figure 4

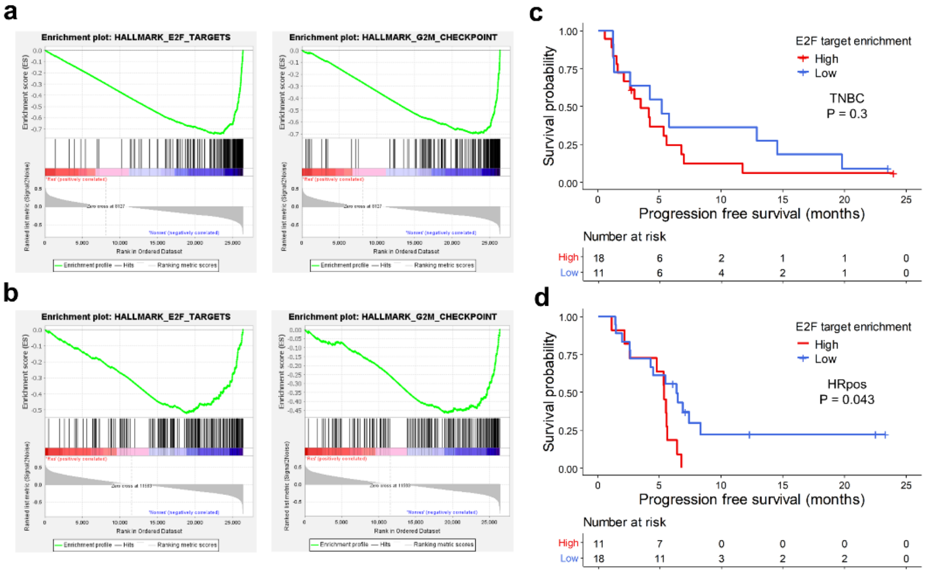

Supplementary Figure 4. Cell cycle associated signature analysis

Gene set enrichment analysis results in (a) HRpos patients; (b) TNBC patients comparing PFS6-responders and PFS6-nonresponders, showing E2F target and G2M checkpoint pathways are significantly enriched in PFS6-nonresponders. (c) Kaplan-Meier curves showing PFS according to E2F targets enrichment scores in the TNBC and (d) HRpos patients.

Supplementary Figure 5

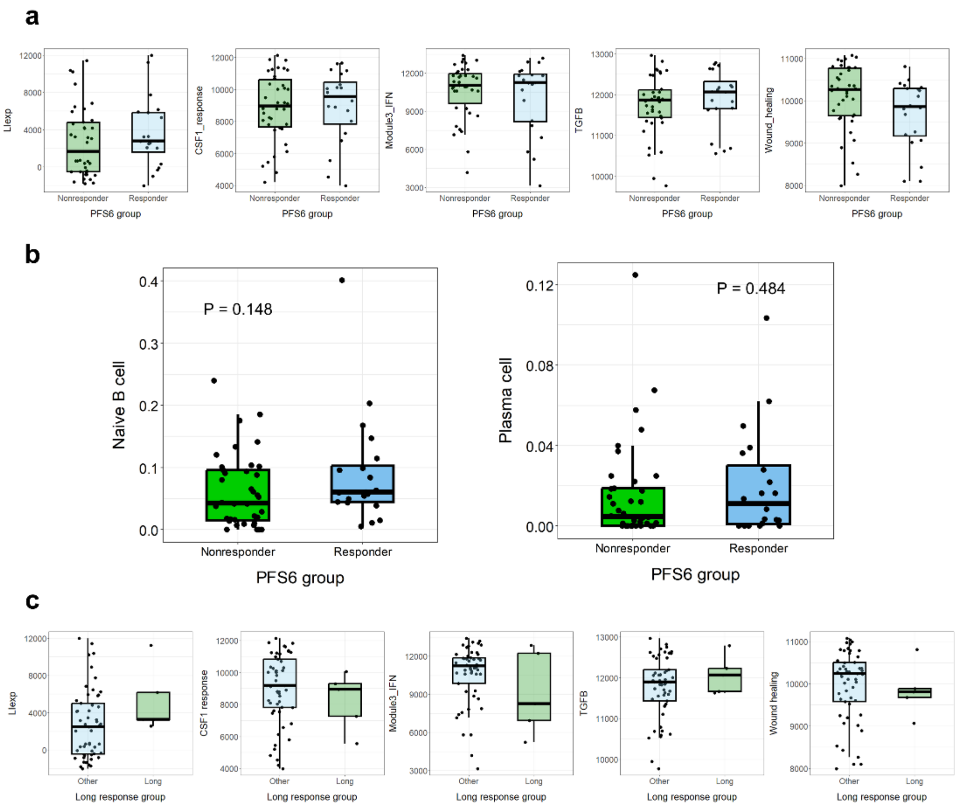

Supplementary Figure 5. Immune-related signatures and PFS6 response

(a) Boxplots showing enrichment scores of major immune-related signatures according to PFS6 groups (b) Box plots showing the naïve B-cell and plasma cell infiltration proportions according to the PFS6 groups. Each dot represents each patient. The p-value is shown. (c) Boxplots showing enrichment scores of major immune-related signatures comparing between long responders and others.

SUPPLEMENTARY TABLES

Supplementary Table 1. Mutation results

Sample id	chrNum	Gene	tumorVaf	hgvsPcode
128-01	chr17	TP53	0.104	p.Thr170ProfsTer4
128-03	chr17	TP53	0.695	p.Arg213Ter
128-03	chr3	FANCD2	0.212	
128-04	chr3	PIK3CA	0.755	p.His1047Arg
128-04	chr6	ESR1	0.211	p.Tyr537Ser
128-04	chr7	KMT2C	0.18	p.Ala1685Ser
128-04	chr17	TP53	0.627	p.Arg196Ter
128-04	chr3	FANCD2	0.174	
128-04	chr10	GATA3	0.31	p.Thr323AspfsTer30
128-05	chr17	TP53	0.532	p.Tyr220Cys
128-05	chr3	PIK3CA	0.313	p.Glu542Lys
128-05	chr5	RAD50	0.0504	p.Asn934IlefsTer6
128-06	chr9	NOTCH1	0.063	p.Ser2486ArgfsTer103
128-06	chr17	TP53	0.588	p.Pro75GlyfsTer65
128-07	chr17	TP53	0.71	p.Arg248Trp
128-07	chr2	BARD1	0.155	p.Glu301AlafsTer13
128-07	chr12	TBX3	0.149	
128-08	chr17	TP53	0.299	p.Val172Phe
128-09	chr17	TP53	0.4	p.Arg282Trp
128-09	chr6	HLA-C	0.105	
128-09	chr6	HLA-B	0.174	
128-01	chr17	TP53	0.803	p.Tyr107Ter
128-01	chr2	DNMT3A	0.786	p.Tyr623ThrfsTer28
128-01	chr19	ERCC2	0.0417	p.Leu183AlafsTer24
128-11	chr17	TP53	0.41	p.Gly262Val
128-11	chr17	ERBB2	0.659	p.Val777Leu
128-11	chr3	PIK3CA	0.311	p.His1047Arg
128-11	chr3	TGFBR2	0.0637	p.Lys153SerfsTer35
128-14	chr16	GRIN2A	0.28	p.Trp1014Ter
128-14	chr17	TP53	0.508	p.Arg342Ter
128-15	chr3	PIK3CA	0.275	p.His1047Leu
128-15	chr17	TP53	0.631	p.Cys124LeufsTer25
128-16	chr17	TP53	0.525	p.Arg248Gln
128-18	chr3	PIK3CA	0.149	p.His1047Arg
128-18	chr5	MAP3K1	0.101	p.Gln1424Ter
128-20	chr9	PAX5	0.0583	p.Ala322LeufsTer11

128-20	chr19	BBC3	0.0667	p.Gly242AlafsTer48
128-21	chr12	KRAS	0.323	p.Gly12Asp
128-21	chr17	TP53	0.6	p.His179Tyr
128-21	chr9	GNAQ	0.0417	p.Thr96Ser
128-21	chr3	TGFBR2	0.0933	p.Lys153SerfsTer35
128-22	chr10	GATA3	0.268	p.Pro409AlafsTer99
128-22	chr10	PTEN	0.227	p.Thr319Ter
128-22	chr20	ASXL1	0.0721	p.Gly645ValfsTer58
128-23	chr3	PIK3CA	0.458	p.His1047Arg
128-23	chr6	ESR1	0.515	p.Glu380Gln
128-23	chr1	ARID1A	0.226	p.Pro989ValfsTer51
128-24	chr6	ESR1	0.69	p.Leu536Pro
128-24	chr7	KMT2C	0.09	p.Arg904Ter
128-24	chr9	GNAQ	0.105	p.Thr96Ser
128-24	chr7	KMT2C	0.167	
128-25	chr17	TP53	0.442	p.Leu252_Ile254del
129-01	chr17	TP53	0.222	p.Gly245Ser
129-01	chr3	PIK3CA	0.367	p.Glu542Lys
129-01	chr17	NF1	0.169	p.Tyr2285ThrfsTer5
129-02	chr6	ESR1	0.209	p.Tyr537Asn
129-02	chr9	GNAQ	0.0548	p.Thr96Ser
129-02	chr10	GATA3	0.359	
129-03	chr7	EZH2	0.0678	p.Pro214HisfsTer27
129-03	chr17	TP53	0.833	p.Ser241ProfsTer6
129-04	chr9	GNAQ	0.114	p.Thr96Ser
129-04	chr9	TSC1	0.108	
129-04	chr17	TP53	0.374	p.Asp281_Leu289delinsVal
129-05	chr17	TP53	0.371	p.Cys135Phe
129-05	chr3	PIK3CA	0.465	p.His1047Arg
129-06	chr1	BCL10	0.0651	p.Ile46TyrfsTer24
129-06	chr5	PIK3R1	0.758	p.Leu609IlefsTer54
129-06	chr16	CREBBP	0.414	p.Gln442LysfsTer28
129-06	chr17	TP53	0.675	p.Thr150_Pro151dup
129-09	chr17	TP53	0.214	p.Cys141Tyr
129-09	chr3	FANCD2	0.103	
129-09	chrX	AR	0.126	p.Leu57Gln
129-09	chr5	PIK3R1	0.16	p.Asn344MetfsTer29
129-09	chr7	KMT2C	0.338	
129-10	chr9	TGFBR1	0.294	

129-10	chr7	KMT2C	0.33	
129-10	chr17	TP53	0.19	p.Gly59ValfsTer64
130-02	chr17	TP53	0.43	p.Arg273His
130-04	chr15	SMAD3	0.36	p.Arg243His
130-04	chr17	ACTG1	0.127	
130-06	chr6	ESR1	0.266	p.Tyr537Asn
130-09	chr17	TP53	0.5	p.Phe109TrpfsTer41
130-10	chr3	PIK3CA	0.327	p.Met1043Ile
130-10	chr6	ESR1	0.385	p.Tyr537Ser
132-01	chr14	AKT1	0.551	p.Glu17Lys
132-01	chr17	TP53	0.396	p.Ser241Phe
132-01	chr9	GNAQ	0.177	p.Thr96Ser
142-02	chr1	FH	0.225	p.Ser11Ter
142-02	chr11	ATM	0.0541	
142-02	chr17	TP53	0.386	p.Arg213Ter
142-02	chr9	GNAQ	0.167	p.Thr96Ser
166-01	chr14	AKT1	0.117	p.Glu17Lys
166-01	chr16	ANKRD11	0.0166	p.Lys369Arg
166-01	chr6	ESR1	0.109	p.Asp538Gly
166-01	chr10	GATA3	0.103	p.Ter445LeufsTer63
166-02	chr17	TP53	0.387	p.Tyr163Cys
166-02	chr22	NF2	0.325	p.Gln470Ter
170-01	chr17	TP53	0.0378	p.Tyr163Cys
170-01	chr11	INPPL1	0.188	p.Gly12AlafsTer15
170-02	chr6	ESR1	0.619	p.Asp538Gly
170-03	chr13	LATS2	0.153	p.Pro479_Ala480dup
170-03	chr14	FOXA1	0.2	p.Ala353GlufsTer9
170-03	chr17	TP53	0.0789	p.Ser362AlafsTer8
170-04	chr17	TP53	0.154	p.Arg175His
170-04	chr3	PIK3CA	0.154	p.His1047Arg
170-05	chr17	TP53	0.155	p.Cys242Trp
170-05	chr9	NOTCH1	0.0851	p.Ile870ThrfsTer301
170-06	chr1	MUTYH	0.0533	p.Gln264Ter
170-06	chr17	TP53	0.239	p.Cys135Trp
170-06	chr7	EGFR	0.0361	p.Arg521Lys
170-06	chr9	GNAQ	0.265	p.Thr96Ser
170-06	chr1	ERRFI1	0.0629	p.Cys146Ter
170-06	chr10	TCF7L2	0.108	p.Cys469ValfsTer8
170-06	chr12	EP400	0.0368	p.Thr2552ProfsTer43

170-06	chr19	KMT2B	0.0508	p.Pro2138ArgfsTer15
H14-01	chr17	TP53	0.262	p.Gly245Asp
H14-01	chr9	GNAQ	0.0577	p.Thr96Ser
H14-01	chr13	LATS2	0.1	p.Pro479_Ala480dup
H14-02	chr17	TP53	0.58	p.Leu130His
H14-02	chr17	NF1	0.603	p.Gln1235Ter
H14-02	chr2	INHA	0.0976	
H14-02	chr3	PIK3CA	0.743	p.Glu545Lys
H14-02	chr7	KMT2C	0.322	p.Ser1824Ter
H14-02	chr1	ARID1A	0.346	p.Gly1644TrpfsTer4
H14-02	chr5	MAP3K1	0.331	p.Met472IlefsTer9
H14-02	chr5	MAP3K1	0.282	p.Ser696Ter
H14-03	chr17	TP53	0.174	p.Gln144His
H14-03	chr17	TP53	0.17	p.Gln144Pro
H14-03	chr9	GNAQ	0.241	p.Thr96Ser
H14-03	chr10	GATA3	0.113	p.Pro409AlafsTer99
H14-05	chr16	GRIN2A	0.133	p.Ser944Ter
H14-05	chr3	PIK3CA	0.11	p.His1047Arg
H14-05	chr6	ESR1	0.291	p.Glu380Gln
H14-05	chr7	KMT2C	0.107	p.Arg904Ter
H14-05	chr9	GNAQ	0.0755	p.Thr96Ser
H14-05	chr17	TP53	0.244	p.Ser241ProfsTer6
H14-06	chr3	PIK3CA	0.184	p.His1047Arg
H14-06	chr3	TGFBR2	0.0526	p.Lys153SerfsTer35
H14-06	chr10	ARID5B	0.0195	p.Lys1027ArgfsTer8
H14-06	chr13	LATS2	0.2	p.Pro479_Ala480dup
H14-06	chr17	TP53	0.434	p.Gly59ArgfsTer2
H14-08	chr11	ATM	0.111	p.Ala59Asp
H14-08	chr11	KMT2A	0.136	p.Cys1189Ter
H14-08	chr13	BRCA2	0.161	p.Ser942Ter
H14-08	chr5	MSH3	0.0678	
H14-08	chr5	RAD50	0.1	
H14-08	chr6	ESR1	0.329	p.Asp538Gly
H14-08	chr9	GNAQ	0.17	p.Thr96Ser
H14-08	chr9	KLF4	0.0638	p.Glu323Ter
H14-09	chr17	TP53	0.0733	p.Gln192Ter
H14-11	chr11	KMT2A	0.235	
H14-11	chr17	TP53	0.448	p.Arg337Leu
H14-11	chr17	RNF43	0.0968	p.Tyr89Ter

H14-11	chr19	CIC	0.0779	p.Arg1512Leu
H14-11	chr3	PIK3CA	0.853	p.Glu542Lys
H14-11	chr3	PIK3CA	0.868	p.Glu726Lys
H14-11	chr6	HLA-A	0.222	
H14-11	chr6	EPHA7	0.148	
H14-11	chr7	KMT2C	0.222	p.Arg904Ter
H14-11	chr9	GNAQ	0.258	p.Thr96Ser
H14-11	chr6	ARID1B	0.0674	p.Gly734AlafsTer11
H14-11	chr8	ESCO2	0.174	p.Thr254AsnfsTer27
H14-11	chr13	LATS2	0.0758	p.Pro479_Ala480dup
H14-12	chr1	SESN2	0.0782	p.Glu135Ter
H14-12	chr17	TP53	0.0909	
H14-12	chr3	PIK3CA	0.0611	p.His1047Arg
H14-12	chr1	SPEN	0.0128	p.Arg986AlafsTer24
H14-13	chr5	SDHA	0.393	p.Leu649GlufsTer4
H14-13	chr17	TP53	0.633	p.Asp148IlefsTer22
H14-14	chr17	TP53	0.0576	p.Tyr220Cys
H14-14	chr17	NF1	0.0684	p.Gln1399Ter
H14-14	chr3	PIK3CA	0.0571	p.Cys378Phe
H14-14	chr3	MLH1	0.0741	p.Leu348AlafsTer21
H14-16	chr17	ERBB2	0.236	p.Asp769Tyr
H14-17	chr1	ARID1A	0.705	p.Gln379Ter
H14-17	chr11	MRE11A	0.0833	
H14-17	chr12	ARID2	0.294	p.Gln1100Ter
H14-17	chr3	FOXP1	0.387	
H14-17	chr7	KMT2C	0.103	p.Cys391Ter
H14-17	chr1	BCL10	0.0851	p.Ile46TyrfsTer24
H14-17	chr5	PIK3R1	0.143	p.Arg577_Leu581del
H14-17	chr16	CBFB	0.531	
H14-17	chr16	CDH1	0.628	p.Phe486LeufsTer31
H14-17	chr17	NF1	0.546	p.Phe1708LeufsTer2
H14-18	chr17	TP53	0.631	p.Pro278Arg
H14-18	chr9	CDKN2A	0.545	p.Tyr44Ter
H14-19	chr12	KMT2D	0.111	p.Gln3919Ter
H14-19	chr3	MLH1	0.0842	p.Val384Asp
H14-19	chr3	MLH1	0.0882	
H14-19	chr6	CDKN1A	0.0479	p.Ser31Arg
H14-19	chr9	GNAQ	0.0833	p.Thr96Ser
H14-20	chr3	PIK3CA	0.288	p.Glu545Lys

H14-20	chr15	TP53BP1	0.0877	
H14-21	chr14	AKT1	0.844	p.Glu17Lys
H14-21	chr9	CDKN2A	0.67	p.Arg80Ter
H14-21	chr6	HLA-A	0.303	p.Asp251ThrfsTer46
H14-21	chr10	BMPR1A	0.0575	
H14-21	chr11	BIRC3	0.0526	p.Cys572LeufsTer5
H14-21	chr19	ARID3A	0.111	p.Gly37AlafsTer87
H16-01	chr1	BCL10	0.0741	p.Ile46TyrfsTer24
H16-01	chr5	MSH3	0.0741	p.Asn385GlnfsTer19
H16-01	chr5	RAD50	0.103	p.Asn934IlefsTer6
H16-01	chr17	TP53	0.676	p.His115AlafsTer34
H16-02	chr9	GNAQ	0.128	p.Thr96Ser
H16-02	chr17	TP53	0.37	p.Gly302ArgfsTer4
H16-02	chr22	EP300	0.0431	p.Gln577IlefsTer23
H16-03	chr3	PIK3CA	0.0588	p.His1047Arg
H16-03	chr5	NSD1	0.0508	p.Ser2693Ter
H16-05	chr17	TP53	0.302	p.Ile195Phe
H16-06	chr1	PIK3R3	0.0563	p.Arg309Ter
H16-06	chr14	AKT1	0.609	p.Glu17Lys
H16-06	chr4	FBXW7	0.0784	p.Gln242Ter
H16-06	chr15	BLM	0.0741	p.Asn515MetfsTer16
H16-07	chr17	TP53	0.905	p.Pro278Leu
H16-07	chr9	GNAQ	0.182	p.Thr96Ser
H16-07	chr16	ANKRD11	0.0602	p.Pro1926ArgfsTer37
H16-08	chr15	TP53BP1	0.233	p.Glu1170Ter
H16-08	chr7	KMT2C	0.151	p.Leu762Ter
H16-08	chr9	GNAQ	0.262	p.Thr96Ser
H16-09	chr14	AKT1	0.439	p.Glu17Lys
H16-09	chr17	TP53	0.454	
H16-09	chr17	NF1	0.393	
H16-09	chr18	SMAD4	0.458	p.Gln83Ter
H16-09	chr4	REST	0.469	p.Glu167Ter
H16-09	chr7	BRAF	0.365	p.Gly466Ala
H16-09	chr9	GNAQ	0.107	p.Thr96Ser
H16-09	chr11	MEN1	0.0247	
H16-09	chr16	ZFHX3	0.137	p.Ser3596ArgfsTer70
H16-10	chr14	AKT1	0.212	p.Glu17Lys
H16-10	chr17	TP53	0.124	p.Glu285Lys
H16-11	chrX	BCORL1	0.116	p.Gln856Ter

H16-11	chr17	MAP2K4	0.471	p.Cys382ValfsTer31
H16-13	chr17	TP53	0.163	p.Val157Phe
H16-13	chr15	BLM	0.066	p.Asn515LysfsTer2
H16-14	chr17	TP53	0.177	p.Gly266Val
H16-14	chr9	GNAQ	0.109	p.Thr96Ser
H16-14	chrX	KDM5C	0.0778	p.Glu465ArgfsTer52
H16-14	chr8	ESCO2	0.105	p.Thr254AsnfsTer27
H16-14	chr10	PTEN	0.148	p.Lys13ProfsTer2
H16-16	chr13	BRCA2	0.325	p.Asn372His
H16-16	chr6	HLA-A	0.781	p.Leu180Trp
H16-16	chr6	HLA-A	0.184	p.Trp228Ter
H16-16	chr7	PMS2	0.158	p.Gly857Ala
H16-16	chr7	KMT2C	0.253	p.Ala1685Ser
H16-16	chr9	GNAQ	0.192	p.Thr96Ser
H16-16	chrX	AR	0.273	p.Leu57Gln
H16-16	chr5	SDHA	0.0917	p.Leu649GlufsTer4
H16-16	chr10	GATA3	0.368	p.Pro409AlafsTer99
H16-16	chr10	TCF7L2	0.0777	p.Cys469ValfsTer8
H16-17	chr17	TP53	0.342	p.Val272Met
H16-17	chr3	PIK3CA	0.297	p.Glu545Lys
H16-17	chr9	GNAQ	0.0952	p.Thr96Ser
H16-17	chr7	KMT2C	0.267	
H16-17	chr10	PTEN	0.395	p.Glu242Ter
H16-17	chr16	ZFHX3	0.117	p.Ser3596ArgfsTer70
H16-18	chr16	ZFHX3	0.439	p.Ser2600Ter
H16-18	chr17	TP53	0.35	
H16-18	chr3	PIK3CA	0.554	p.His1047Arg
H16-18	chr7	BRAF	0.31	p.Gly469Ala
H16-18	chr9	GNAQ	0.321	p.Thr96Ser
H16-18	chrX	AR	0.302	p.Leu57Gln
H16-18	chr12	RECQL	0.0984	p.Val41SerfsTer14
H16-18	chr14	MAX	0.427	p.Lys104ArgfsTer66
H16-20	chr14	AKT1	0.474	p.Glu17Lys
H16-20	chr17	TP53	0.484	p.Pro151Ser
H16-20	chr7	KMT2C	0.0654	p.Arg904Ter
H16-20	chr9	GNAQ	0.169	p.Thr96Ser
H16-20	chr13	DIS3	0.023	p.Val105TyrfsTer8

Supplementary Table 2. Single gene mutation association with PFS6 rate or PFS

Gene	Fisher test p-value for PFS6 rate	Log-rank test p-value for PFS	Frequency
TP53	0.037	0.045	53
GNAQ	0.794	0.602	22
PIK3CA	1.000	0.920	20
KMT2C	1.000	0.732	12
ESR1	0.484	0.808	10
AKT1	0.704	0.826	7
GATA3	0.664	0.927	6
NF1	0.059	0.215	5
LATS2	1.000	0.959	4
AR	1.000	0.239	3
ARID1A	0.551	0.282	3
BCL10	1.000	0.341	3
FANCD2	0.293	0.227	3
HLA.A	1.000	0.658	3
PIK3R1	1.000	0.549	3
PTEN	0.293	0.416	3
RAD50	1.000	0.539	3
TGFBR2	0.293	0.013	3
ZFHX3	1.000	0.591	3
ANKRD11	1.000	0.723	2
ATM	1.000	0.376	2
BLM	1.000	0.098	2
BRAF	1.000	0.989	2
BRCA2	0.133	0.807	2
CDKN2A	0.528	0.437	2
ERBB2	1.000	0.367	2
ESCO2	0.528	0.543	2
GRIN2A	1.000	0.939	2
KMT2A	1.000	0.881	2
MAP3K1	1.000	0.848	2
MLH1	1.000	0.916	2
MSH3	1.000	0.938	2
NOTCH1	0.528	0.172	2
SDHA	1.000	0.589	2
TCF7L2	1.000	0.965	2
TP53BP1	0.133	0.123	2
ACTG1	1.000	0.019	1

ARID1B	1.000	0.785	1
ARID2	0.368	0.122	1
ARID3A	1.000	0.130	1
ARID5B	1.000	0.724	1
ASXL1	1.000	0.523	1
BARD1	1.000	0.360	1
BBC3	1.000	0.510	1
BCORL1	0.368	0.704	1
BIRC3	1.000	0.130	1
BMPR1A	1.000	0.130	1
CBFB	0.368	0.122	1
CDH1	0.368	0.122	1
CDKN1A	0.368	0.555	1
CIC	1.000	0.785	1
CREBBP	1.000	0.693	1
DIS3	1.000	0.633	1
DNMT3A	0.368	0.261	1
EGFR	1.000	0.755	1
EP300	0.368	0.122	1
EP400	1.000	0.755	1
EPHA7	1.000	0.785	1
ERCC2	0.368	0.261	1
ERRFI1	1.000	0.755	1
EZH2	1.000	0.041	1
FBXW7	1.000	0.567	1
FH	1.000	0.000	1
FOXA1	1.000	0.567	1
FOXP1	0.368	0.122	1
HLA.B	1.000	0.089	1
HLA.C	1.000	0.089	1
INHA	0.368	0.807	1
INPPL1	1.000	0.479	1
KDM5C	1.000	0.541	1
KLF4	0.368	0.969	1
KMT2B	1.000	0.755	1
KMT2D	0.368	0.555	1
KRAS	1.000	0.000	1
MAP2K4	0.368	0.704	1
MAX	1.000	0.029	1

MEN1	0.368	0.516	1
MRE11A	0.368	0.122	1
MUTYH	1.000	0.755	1
NF2	1.000	0.390	1
NSD1	0.368	0.122	1
PAX5	1.000	0.510	1
PIK3R3	1.000	0.567	1
PMS2	0.368	0.773	1
RECQL	1.000	0.029	1
REST	0.368	0.516	1
RNF43	1.000	0.785	1
SESN2	0.368	0.937	1
SMAD3	1.000	0.019	1
SMAD4	0.368	0.516	1
SPEN	0.368	0.937	1
TBX3	1.000	0.360	1
TGFBR1	1.000	0.055	1
TSC1	0.368	0.670	1
